# A CT-based nomogram established for differentiating gastrointestinal heterotopic pancreas from gastrointestinal stromal tumor: compared with a machine-learning model

**DOI:** 10.1186/s12880-023-01094-3

**Published:** 2023-09-15

**Authors:** Na Feng, Hai-Yan Chen, Xiao-Jie Wang, Yuan-Fei Lu, Jia-Ping Zhou, Qiao-Mei Zhou, Xin-Bin Wang, Jie-Ni Yu, Ri-Sheng Yu, Jian-Xia Xu

**Affiliations:** 1 Department of Radiology, The Second Affiliated Hospital, Zhejiang University School of Medicine, Jiefang Road 88#, 310009 Hangzhou, China; 2 Department of Radiology, Zhejiang Cancer Hospital, Institute of Basic Medicine and Cancer (IBMC), Chinese Academy of Sciences, 310022 Hangzhou, Zhejiang China; 3 Department of Radiology, The First People’s Hospital of Xiaoshan District, Hangzhou, China; 4 Department of Radiology, The Second Affiliated Hospital, Zhejiang Chinese Medical University, Chaowang Road 318#, 310005 Hangzhou, China

**Keywords:** Heterotopic pancreas, Gastrointestinal stromal tumor, Nomogram, Random Forest, Computed tomography

## Abstract

**Objective:**

To identify CT features and establish a nomogram, compared with a machine learning-based model for distinguishing gastrointestinal heterotopic pancreas (HP) from gastrointestinal stromal tumor (GIST).

**Materials and methods:**

This retrospective study included 148 patients with pathologically confirmed HP (n = 48) and GIST (n = 100) in the stomach or small intestine that were less than 3 cm in size. Clinical information and CT characteristics were collected. A nomogram on account of lasso regression and multivariate logistic regression, and a RandomForest (RF) model based on significant variables in univariate analyses were established. Receiver operating characteristic (ROC) curve, mean area under the curve (AUC), calibration curve and decision curve analysis (DCA) were carried out to evaluate and compare the diagnostic ability of models.

**Results:**

The nomogram identified five CT features as independent predictors of HP diagnosis: age, location, LD/SD ratio, duct-like structure, and HU lesion/pancreas A. Five features were included in RF model and ranked according to their relevance to the differential diagnosis: LD/SD ratio, HU lesion/pancreas A, location, peritumoral hypodensity line and age. The nomogram and RF model yielded AUC of 0.951 (95% CI: 0.842–0.993) and 0.894 (95% CI: 0.766–0.966), respectively. The DeLong test found no statistically significant difference in diagnostic performance (p > 0.05), but DCA revealed that the nomogram surpassed the RF model in clinical usefulness.

**Conclusion:**

Two diagnostic prediction models based on a nomogram as well as RF method were reliable and easy-to-use for distinguishing between HP and GIST, which might also assist treatment planning.

## Keypoints

A nomogram and RandomForest model based on CT images were established for differentiating gastrointestinal heterotopic pancreas from small (≤ 3 cm) GIST.

Age, location, LD/SD ratio, HU lesion/pancreas A and duct-like structure (or peritumoral hypodensity line) were identified as independent predictors of gastrointestinal HP in the models.

Two models both had excellent discriminative performance, despite the nomogram may result in a larger net benefit.

## Introduction

Heterotopic pancreas (HP), also known as “ectopic pancreas”, is defined as pancreatic tissue lacking anatomic or vascular continuity with the main body of the gland [1, 2]. Although HPs can be up to 5.0 cm in size, almost 80% lesions are smaller than 3 cm [3–6]. They were often incidentally found on the upper gastrointestinal system, specifically the stomach, duodenum, and proximal jejunum in around 0.5–13.7% of corpses or 0.2–0.9% of abdominal surgeries [3, 4, 7]. In fact, the true prevalence of HP is difficult to assess as most patients are asymptomatic [8]. Invasive operations for a confirmative diagnosis as well as surgery are generally not recommended [9, 10]. But some larger lesions, or lesions in specific locations (e.g., duodenum papilla), could cause complications similar to those of the normal pancreas such as pancreatitis, pancreatic pseudocysts and even malignancy, or symptoms such as abdominal pain, bleeding and obstruction [1, 8, 11]. Additionally, malignant transformation of HP is extremely rare, and there are only sporadic case reports available in the literature [1, 12, 13].

Since HP more often manifests as a gastrointestinal submucosal lesion, it might be empirically misdiagnosed as gastrointestinal mesenchymal tumors (GIST), the true and most common submucosal tumor [14, 15]. Unlike HP, the biological behavior of GIST is complex and demonstrates varying malignant potential, including recurrences and metastasis [16–18]. Surgery for GISTs was more focused on the risk class rather than just the size or location of the entities [18, 19], and patients often choose regular follow-up visits or surgeries.

In terms of radiologic appearance, gastrointestinal HP and GIST have many overlapping features, and smaller lesions are harder to identify [2, 20]. Computed tomography (CT), as a non-invasive and common imaging examination method, has been emphasized for preoperative diagnosis of HP lesions and differentiation it from GIST [2, 4, 9, 11, 20–23]. Furthermore, the application of artificial intelligence, machine learning and deep learning in radiological imaging has been progressively investigated so far [24–26]. However, due to its rarity, CT features of HP have not been extensively examined in these prior researches, the majority of them only provided descriptive analysis, and no studies have been done to distinguish between the two lesions using nomogram or machine learning method.

Therefore, the purpose of this study was to increase awareness of the imaging appearances of gastrointestinal HP and select the optimal model by establishing a nomogram and comparing it with a machine learning-based model to differentiate it from GIST.

## Materials and methods

### Patients

**Fig. 1:**
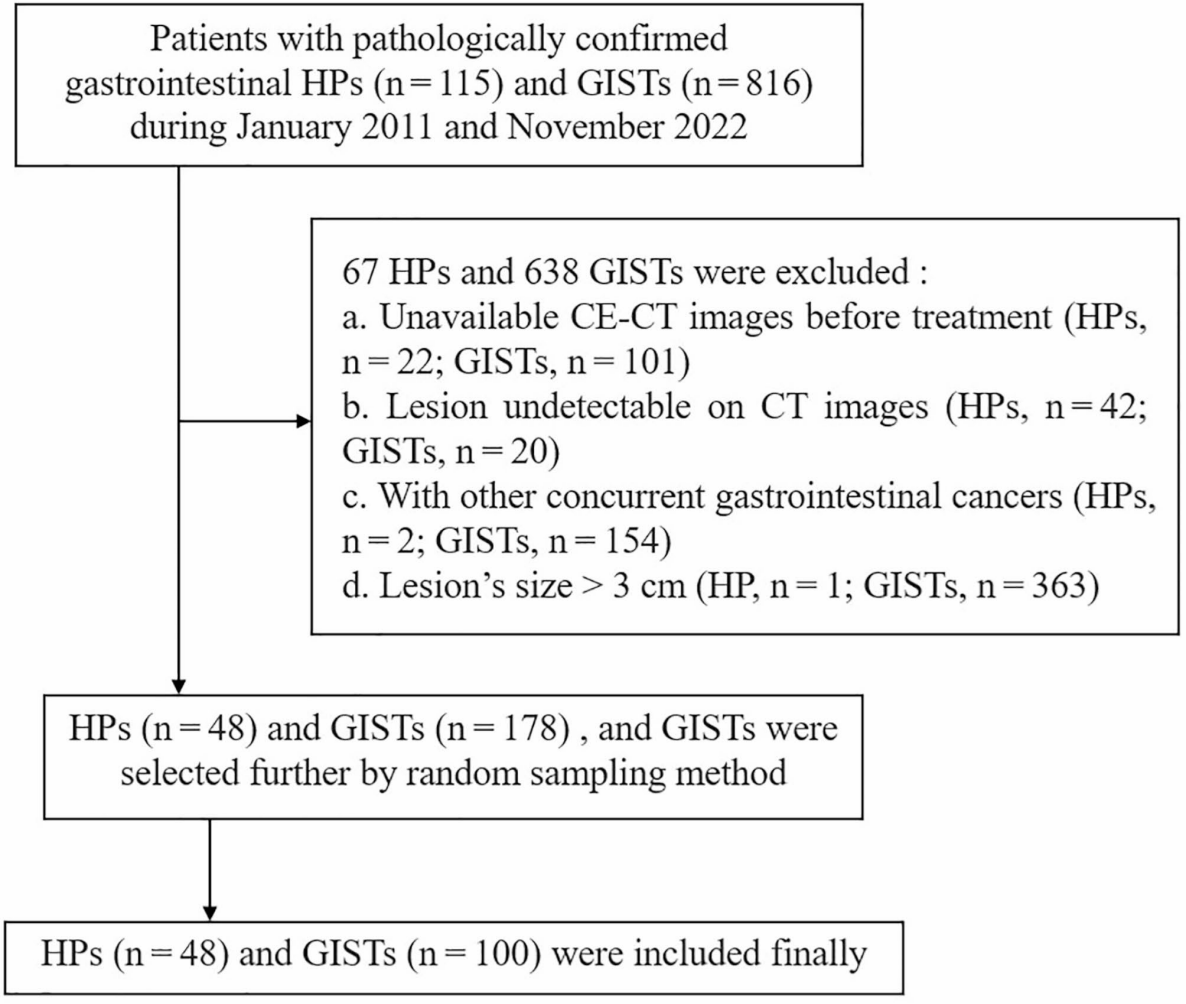
Patient selection and the exclusion criteria of the study

We searched the records between January 2011 and November 2022 in our pathology databases for HP and GIST in the stomach and small bowel by using various combinations of several keywords. All lesions were histopathologically confirmed by surgery or biopsy. We used the following inclusion and exclusion criteria (Fig. 1): (a) patients with HPs or GISTs were confirmed by histopathological diagnosis; (b) patients had detailed clinical data and were available of abdominal CE-CT performed before treatment; (c) CT images with satisfactory quality contained plain phase, arterial phase and portal venous phase; (d) patients without other concurrent gastrointestinal cancers; and (e) lesions ≤ 3 cm in diameter, which was used to avoid potential bias attributed to size differences, because most reported cases with HP had lesion with a long diameter ≤ 3 cm and only one HP of our patients > 3.0 cm in size were excluded. Finally, a total of 148 patients with HPs (n = 48) and GISTs (n = 100) were included. In detail, 37 cases of HPs were from Hospital 1 and 11 cases from Hospital 2, and all 100 cases of GISTs were from Hospital 1. Of the finally enrolled 100 patients with GISTs, 46 had a very low-risk of malignant potential, 42 had low-risk, 7 had intermediate-risk, and 5 had high-risk.

### CT imaging acquisition

Due to the long period of collection, multiple CT scanners were used as follows: TOSHIBA Aquilion 320 (TOSHIBA Medical Systems Corporation), Siemens Somatom Definition AS 6/Flash 64/Perspective (Siemens Medical Systems), Optima CT680 Series/BrightSpeed 16 (GE Medical Systems), and Ingenuity CT 64 (Philips Medical Systems). Enhanced CT images contained plain, arterial, and portal venous phases for all patients. For enhanced images, an automatic power injector was used, and nonionic contrast medium (iopromide/Ultravist 370, Bayer Schering Pharma; Omnipaque 300 g/L, GE Healthcare; 100–120 mL) was administered intravenously at a rate of 3–5 mL/s. Contrast-enhanced CT images were acquired in the arterial phase at 30–40 s and in the portal venous phase at 50–70 s. CT images were obtained at 120 kVp and 150–350 mAs with a 3–5-mm slice thickness and a 320–380-mm field of view.

### Clinical and image analysis

We collect gender, age, chief complaint of all patients. Main chief complaints were classified as digestive tract hemorrhage, abdominal discomfort or pain, both and asymptomatic. Images were analyzed independently by two radiologists (J.X.X. and H.Y.C., with 16 and 5 years of experience in abdominal radiology, respectively) who were blinded to patients’ pathological results. Any disagreements were resolved by consensus after consultation with a third abdominal radiologist (R.S.Y.) with over 30 years of experience.

**Fig. 2:**
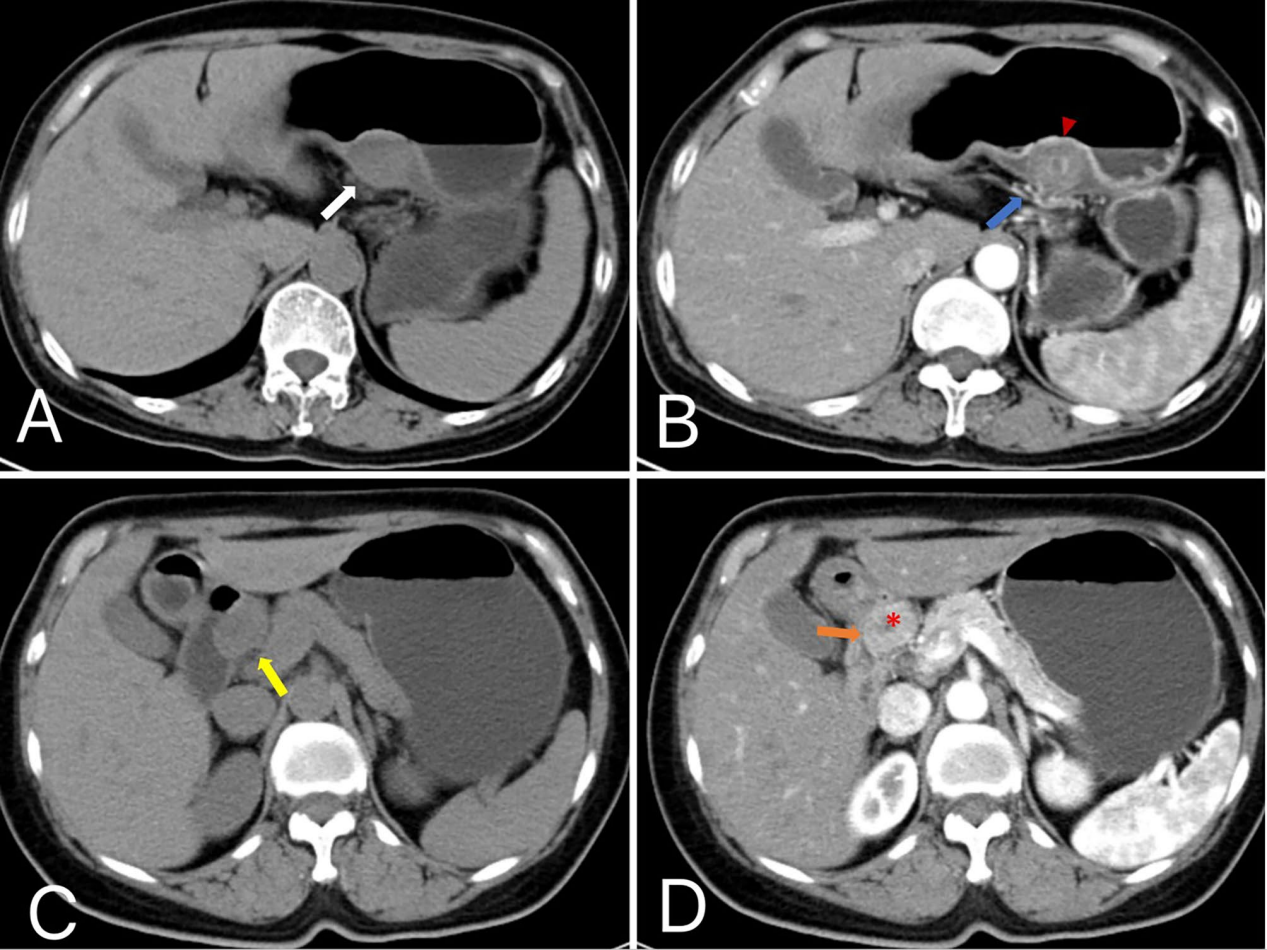
**(A-B)** A GIST located in the lesser curvature of gastric middle body (white arrow), presented as a well-defined ovoid submucosal lesion with mixed growth pattern. The axial image in arterial phase showed enlarged feeding vessel (blue arrow) at the edge of the lesion, and hyperenhancement of the overlying mucosa (arrowhead). **(C-D)** A GIST in the descending part of the duodenum. The axial image showed a well-defined ovoid lesion (yellow arrow) with extraluminal growth pattern. The surface ulceration (*), peritumoral hypodensity line (orange arrow) were presented

For qualitative analysis, main CT items were analyzed as follows: Location (include the upper, middle and lower parts of stomach, and duodenum, jejunum or ileum); contour; imaging type; microlobulated (subtle serrated margin similar to real pancreas); border (well-defined or ill-defined); growth pattern (intraluminal, extraluminal, mixed); peak enhancement phase; enhancement grade; and enhancement pattern. And presence of calcification, surface ulceration (irregular depression of local surface), hyperenhancement of the overlying mucosa (compared with the adjacent normal mucosa), low intralesional attenuation, peritumoral hypodensity line, duct-like structure and EVFDM (enlarged vessels feeding or draining the lesion) (Fig. 2) [23, 27].

**Fig. 3:**
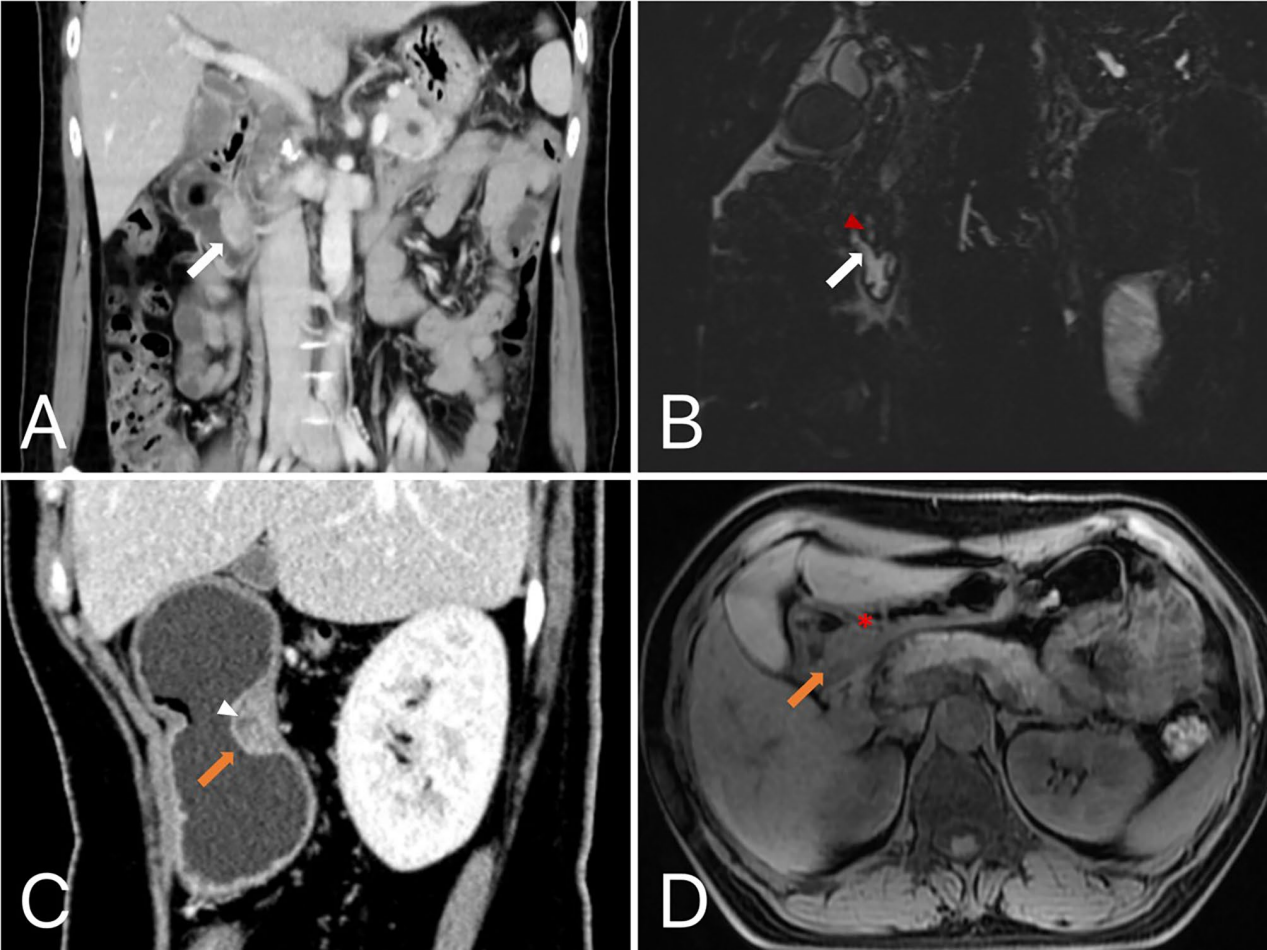
**(A-B)** A HP in duodenal papilla. The coronal image in portal venous phase showed a 3 cm, ovoid and ill-defined lesion (white arrow) with intraluminal growth pattern, presented marked and heterogeneous enhancement totally. A single-opening duct-like structure that opened into the duodenal lumen was showed in the MRCP image (red arrowhead). **(C-D)** A HP located in posterior wall of the gastric antrum. The sagittal CT image in arterial phase showed a 2.6 cm, hill-like submucosal lesion (orange arrow) with intraluminal growth pattern, and demonstrated peritumoral hypodensity line, surface ulceration (white arrowhead). The axial T1-weighted MR image showed similarly a broad- based, microlobulated (*) lesion with isointensity

The stomach location is divided by the lines connecting the trisected points on the lesser and greater curvatures [28]. Lesion contour was classified into round, ovoid (LD/SD ratio of ≤ 1.5), flat (flatter than ovoid and with LD/SD > 1.5), hill-like, or irregular in shape. Low intralesional attenuation was defined as an area with a CT attenuation value < 20 HU in three phases [1, 3]. It was caused by abnormally dilated ducts in HP, but cystic change, hemorrhage and necrosis in GIST could share with this appearance [8, 23]. Imaging type was classified by the content of low intralesional attenuation (solid-dominant, cystic-dominant, mixed). Peritumoral hypodensity line were defined as clear, smooth linear hypodensity demarcation between lesion and gastrointestinal tissue (Fig. 2). Duct-like structure, which also referred to central umbilication in initial studies, was defined as a low-density thin strip-like structure and was relatively easily observed on sagittal or coronal images (Fig. 3) [4, 29]. Enhancement grade and enhancement pattern were assessed on portal venous phase images. And with regard to enhancement grade, difference value < 20 HU was regarded as mild, 20–40 HU as moderate, > 40 HU as strong.

For quantitative analysis, the LD and SD of the lesions were measured first. Mean CT attenuation values of the pancreas and tumors in three phases (HU plain/arterial/venous) were measured using a circular region of interest (ROI) and encompassing as much of the most strongly enhanced section of the lesion as possible. Meanwhile, calcification, hemorrhage, necrosis, cystic degeneration, blood vessels in tumor and adjacent structures were avoid. The pancreatic body-caudal junction site was selected uniformly to measure CT values related to the pancreas. The averages were then used to calculate the enhancement values of tumor: DEAP (HU arterial − HU plain), DEVP (HU venous − HU plain), the enhancement ratio of lesion (HU venous − HU plain/HU plain) and the CT values ratio of lesion to pancreas in plain, arterial, venous phase (HU lesion/pancreas, P/A/V).

### Statistical analysis

Continuous variables are presented as median with standard deviation, compared using the student t test or Mann-Whitney U-test. Categorical variables as the number with a percentage, compared by the chi-square or Fisher’s exact test. Cut-off values for continuous variables were calculated by maximizing the Youden index in the ROC analysis. Besides, during the construction of logistic regression model, some variables were proved to exist multicollinearity, the least absolute shrinkage and selection operator (LASSO) method using the ‘sklearn’ package was employed to select features further. The ‘rms’ package in R software and ‘randomForest’ package in Python were used to construct nomogram and RF model respectively. Calibration of the logistic regression model was assessed by the Hosmer and Lemeshow test for goodness of fit. The RF model was validated using 10-fold cross-validation. The performance and discriminative power of models were assessed with ROC curve, AUC and calibration curve. DCA was performed to evaluate and compare the net benefit of two models. And AUCs between two models were compared by the DeLong test. Statistical significance was defined with a two-sided p-value of < 0.05. SPSS software (ver. 25.0, IBM Inc.), R software (ver. 4.2.2; http://www.R-project.org) and Python programming language (ver. 3.9.0, https://www.python.org) were used to process all data and establish models, besides comparison of ROCs using MedCalc software (ver. 19.8, MedCalc Software bvba).

## Results

### Clinical analysis

Table 1 summarizes the comparison of clinical features. No significant differences were observed in gender and chief complaint. However, age distribution differed significantly between the two groups (HPs = 44.17 ± 13.14 y; GISTs = 60.00 ± 9.46 y, P < 0.001).

**Table 1:** Clinical features of HPs and GISTs

Clinical features	HPs (n = 48)	GISTs (n = 100)	P value*
Age(year)	44.17 ± 13.14	60.00 ± 9.46	***< 0.001***
Gender			0.423
Male	24(50.0%)	43(43.0%)	
Female	24(50.0%)	57(57.0%)	
Chief complaint			0.247
Digestive tract hemorrhage	1(2.1%)	8(8.0%)	
Abdominal discomfort or pain	17(35.4%)	23(23.0%)	
Both	2(4.2%)	3(3.0%)	
Asymptomatic	28(58.3%)	66(66.0%)	

* P values ≤ 0.05 in bold and italics indicated a statistically significant difference between groups

### Qualitative and quantitative image analysis

Table 2 summarizes the comparison of CT imaging features. Location (P < 0.001), border (P < 0.001), peritumoral hypodensity line (P = 0.002), duct-like structure (P = 0.002), enhancement grade (P = 0.004), enhancement pattern (P = 0.008), LD/SD ratio (P = 0.005), HU arterial (P = 0.029), DEAP (P = 0.030), and HU lesion/pancreas A (P = 0.006) showed a statistically significant difference between the two groups.

**Table 2:** CT features comparison among HPs and GISTs: univariate analysis

CT findings	HPs (n = 48)	GISTs (n = 100)	P value*
Location			***< 0.001***
The upper part of stomach	5(10.4%)	49(49.0%)	
The middle part of stomach	6(12.5%)	15(15.0%)	
The lower part of stomach	25(52.1%)	17(17.0%)	
Duodenum	7(14.6%)	10(10.0%)	
Jejunum or ileum	5(10.4%)	9(9.0%)	
Multiple lesions			0.622
No	45(93.7%)	97(97.0%)	
Yes	3(6.3%)	3(3.0%)	
Contour			0.200
Round	7(14.6%)	16(16.0%)	
Ovoid	15(31.3%)	49(49.0%)	
Hill-like	9(18.8%)	10(10.0%)	
Flat	7(14.6%)	8(8.0%)	
Irregular	10(20.8%)	17(17.0%)	
Imaging type			0.096
Solid-dominant	35(72.9%)	79(79.0%)	
Cystic-dominant	5(10.4%)	2(2.0%)	
Mixed	8(16.7%)	19(19.0%)	
Microlobulated			0.193
No	19(39.6%)	51(51.0%)	
Yes	29(60.4%)	49(49.0%)	
Calcification			0.603
No	46(95.8%)	92(92.0%)	
Yes	2(4.2%)	8(8.0%)	
Growth pattern			0.462
Intraluminal	21(43.8%)	44(44.0%)	
Extraluminal	6(12.5%)	20(20.0%)	
Mixed	21(43.8%)	36(36.0%)	
Low intralesional attenuation			0.791
No	37(77.1%)	79(79.0%)	
Yes	11(22.9%)	21(21.0%)	
Border			***< 0.001***
Well-defined	25(52.1%)	84(84.0%)	
Ill-defined	23(47.9%)	16(16.0%)	
Peritumoral hypodensity line			***0.002***
No	40(83.3%)	58(58.0%)	
Yes	8(16.7%)	42(42.0%)	
Duct-like structure			***0.002***
No	41(85.4%)	99(99.0%)	
Yes	7(14.6%)	1(1.0%)	
Surface ulceration			0.097
No	45(93.8%)	84(84.0%)	
Yes	3(6.3%)	16(16.0%)	
EVFDM			0.588
No	29(60.4%)	65(65.0%)	
Yes	19(39.6%)	35(35.0%)	
Hyperenhancement of the overlying mucosa			0.481
No	37(77.1%)	82(82.0%)	
Yes	11(22.9%)	18(18.0%)	
Peak enhancement phase			0.256
Arterial phase	9(18.8%)	13(13.0%)	
Venous phase	20(41.7%)	56(56.0%)	
Both	19(39.6%)	31(31.0%)	
Enhancement grade			***0.004***
Mild	7(14.6%)	8(8.0%)	
Moderate	8(16.7%)	44(44.0%)	
Strong	33(68.8%)	48(48.0%)	
Enhancement pattern			***0.008***
Heterogeneous	13(27.1%)	50(50.0%)	
Homogeneous	35(72.9%)	50(50.0%)	
LD	19.71 ± 6.30	21.62 ± 6.00	0.972
SD	13.69 ± 4.59	16.65 ± 4.87	0.406
LD/SD ratio	1.50(0.52)	1.32(0.28)	***0.005***
HU plain	44.04(13.94)	40.93(8.10)	0.255
HU arterial	86.81(37.48)	67.54(30.43)	***0.029***
HU venous	93.80(26.26)	81.86(29.65)	0.052
DEAP	43.54(34.18)	26.93(32.04)	***0.030***
DEVP	50.35(26.30)	38.72(26.05)	0.116
Enhancement ratio	1.17(0.66)	0.99(0.73)	0.158
HU lesion/pancreas, P	0.88(0.31)	0.84(0.21)	0.399
HU lesion/pancreas, A	0.88(0.31)	0.67(0.20)	***0.006***
HU lesion/pancreas, V	1.03(0.23)	0.90(0.31)	0.077

* P values ≤ 0.05 in bold and italics indicated a statistically significant difference between groups

HU plain/arterial/venous = the CT attenuation value of plain/arterial/portal venous phase; DEAP = HU arterial − HU plain; DEVP = HU venous − HU plain; Enhancement ratio= (HU venous − HU plain)/HU plain of lesion; HU lesion/pancreas, P/A/V = the CT values ratio of lesion to pancreas in plain, arterial, venous phase

Besides, cut-off values for continuous variables were set respectively according to ROC analysis. As a result, age < 53.5 y, LD/SD ratio > 1.47, HU arterial > 83.895 HU, DEAP > 39.675 HU and HU lesion/pancreas A > 0.805 were found to be critical imaging features of HPs for differentiating it from GISTs.

### Establishment of nomogram

**Fig. 4:**
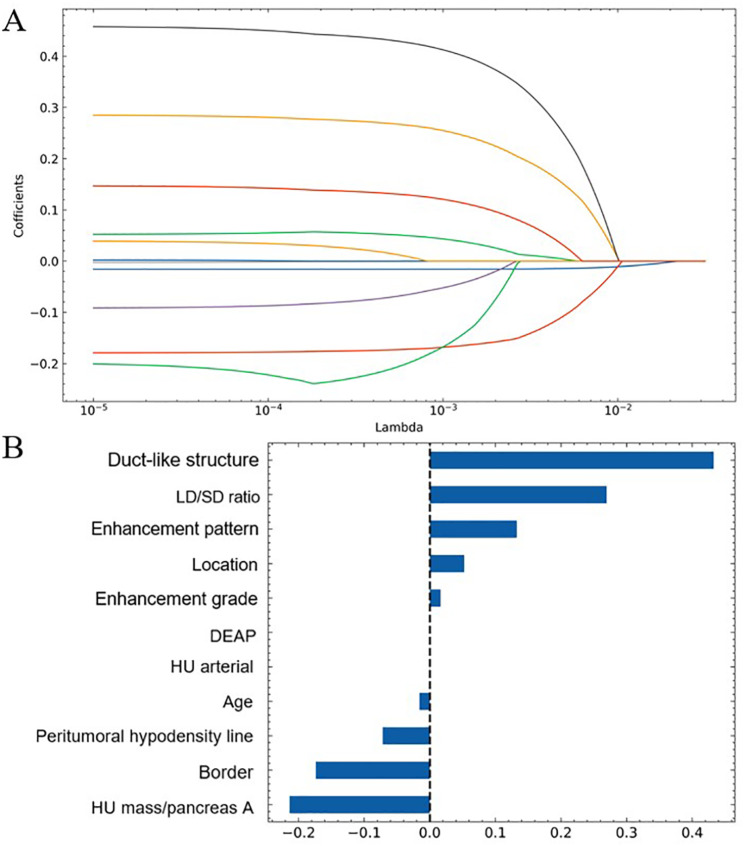
Results of lasso regression. **(A)** shows lasso coefficient profiles of the 11 CT features. **(B)** shows variable importance ranking, and 9 concrete variables were retained with nonzero coefficients

**Fig. 5:**
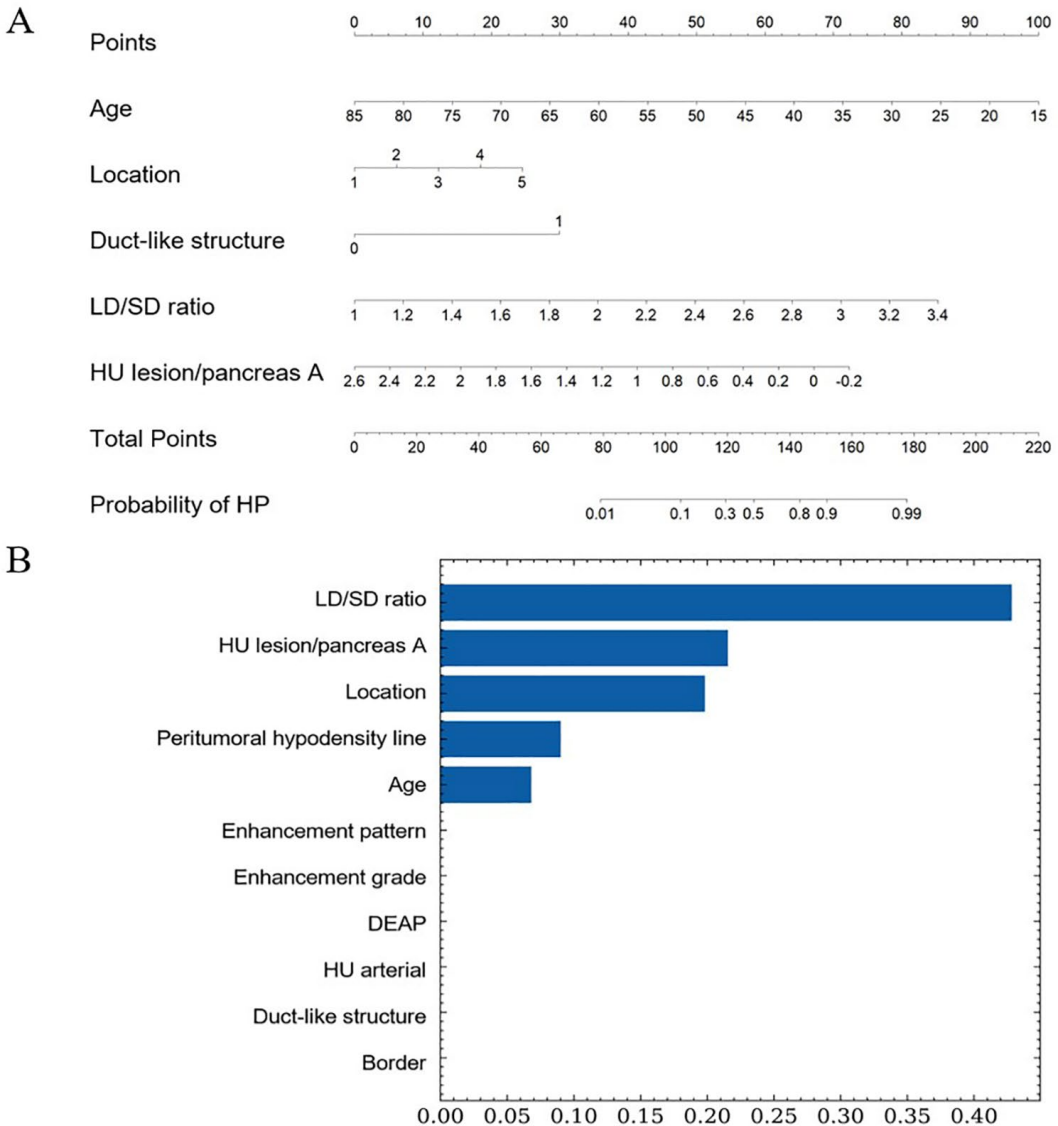
Nomogram **(A)** and RandomForest model **(B)** for differentiating between heterotopic pancreas and gastrointestinal stromal tumor

The lasso regression was used to select features further in order to minimize multicollinearity, and 2 variables were excluded (i.e. HU arterial and DEAP) with zero coefficients (Fig. 4). For further verification, multivariate logistic regression showed 5 independent predictors among the remaining 9 variables for gastrointestinal HPs diagnosis and distinguishing from GISTs (Table 3), which included age (OR, 0.875; 95% CI, 0.835–0.917; P<0.001), location (OR, 1.773; 95% CI, 1.118–2.811; P = 0.015), LD/SD ratio (OR, 27.699; 95% CI, 3.742–205.030; P = 0.001), duct-like structure (OR, 16.411; 95% CI, 1.474–182.762; P = 0.023) and HU lesion/pancreas A (OR, 0.090; 95% CI, 0.011–0.729; P = 0.024). The results of Hosmer-Lemeshow goodness-of-fit test (χ^2^ = 7.813; P = 0.452) indicated great calibration of the logistic regression model. The nomogram obtained at multivariable analysis were displayed in Fig. 5A.

**Table 3:** Multivariate regression analysis for differential diagnosis

Variables	B	P *	OR	95%CI for OR	
				Lower	Upper
Age	-0.133	***< 0.001***	0.875	0.835	0.917
Location	0.573	***0.015***	1.773	1.118	2.811
Duct-like structure	2.798	***0.023***	16.411	1.474	182.762
LD/SD ratio	3.321	***0.001***	27.699	3.742	205.030
HU lesion/pancreas, A	-2.412	***0.024***	0.090	0.011	0.729

* P values < 0.05 in bold and italics indicated a statistically significant difference between groups

### RF model construction

The RF model was established according to the eleven relevant variables in univariate analyses directly. The Gini index was used to judge the importance of different variables in the model (Fig. 5B). Ten-fold cross-validation was performed to evaluate the reliability and reproducibility of the model. Finally, 5 features were retained as independent predictors and ranked according to their relevance to the differential diagnosis as follows: LD/SD ratio, HU lesion/pancreas A, location, peritumoral hypodensity line and age.

### Comparison of model performance

**Fig. 6:**
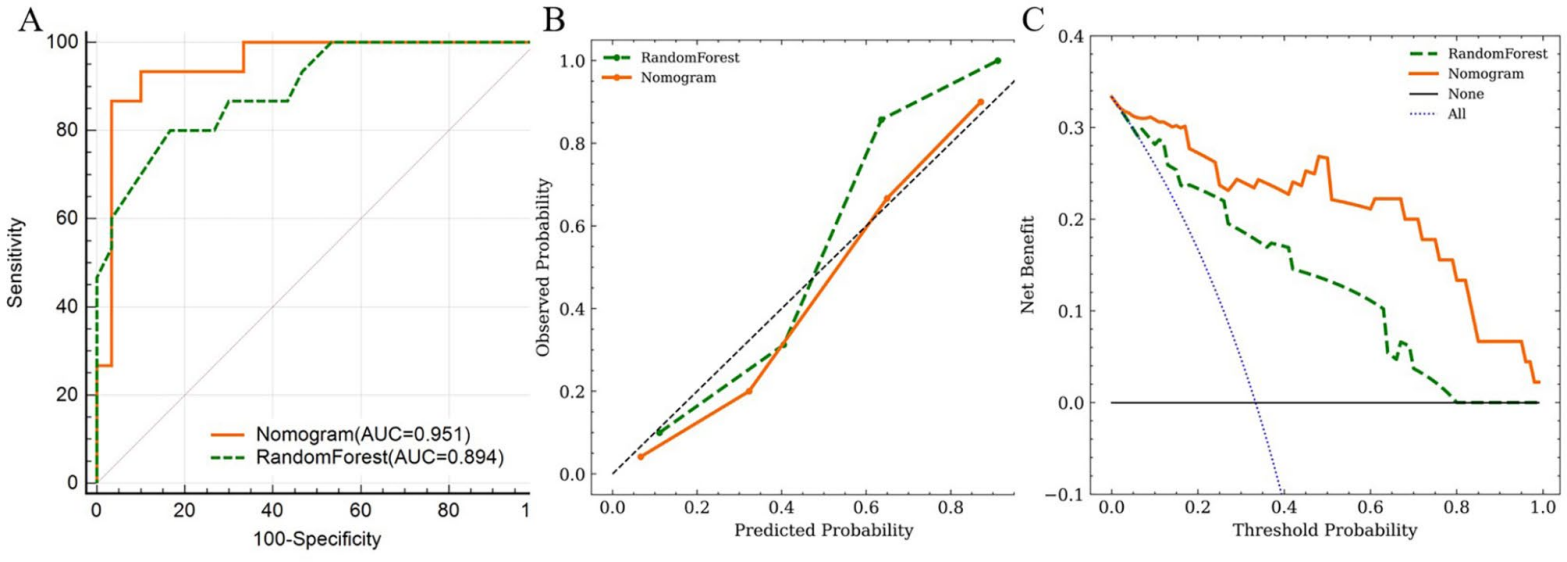
The performance of nomogram and RandomForest model was assessed and compared by receiver operating characteristic curves **(A)** and calibration curves **(B)**. Decision curves **(C)** quantified the probabilities of net benefits at a threshold probability from 0.0 to 1.0. The farther the decision curve is from the two extreme curves, the higher the clinical decision net benefit of the model. The result demonstrated a higher net benefit of the nomogram

The AUCs of the nomogram and RF model were 0.951 (95% CI: 0.842–0.993) and 0.894 (95% CI: 0.766–0.966), respectively (Fig. 6A). In addition, the comparison of ROC curves showed no statistical difference (P = 0.2298) testified by DeLong test. The calibration curves indicated the goodness-of-fit of both models (Fig. 6B). Finally, DCA was conducted to evaluate the clinical utility of two modes, and the result indicated that the nomogram provided a larger net benefit than the RF model (Fig. 6C).

## Discussion

In this study, a nomogram based on logistic regression and a machine learning model exploring RF method was established for the differential diagnosis of gastrointestinal HP and GIST. Among significant independent predictors, age, location, LD/SD ratio and HU lesion/pancreas A were shared by the two models, except for the unique duct-like structure in the nomogram and peritumoral hypodensity line in the RF model. The results confirmed the two models’ good discrimination and calibration, but the nomogram might result in a larger net benefit than the RF model according to decision curve analysis.

HP, which is defined as a kind of ectopic flat glandular tissue and shares similar histological composition with normal pancreas, tends to manifest as broad-based lesions with wall-attached growth pattern, and thus often has a larger LD/SD ratio. But GIST is a true tumor that prefer to grow towards smaller ratio. LD/SD ratio was an important diagnostic predictor in two models with median value of 1.50 and cut-off point of 1.47, which is essentially consistent with many previous studies [2, 4, 22, 30].

As for location, there was no statistically significant difference in the distribution of HP and GIST on the stomach and small intestine. The result of HP is similar to a clinicopathological study [31], in which the stomach (97/184, 52.7%) was the most common location in the gastrointestinal tract, followed by the small intestine (48/184, 26%). However, presumably owing to sample size error, the incidence in the small intestine was significantly higher than in the stomach in other studies, particularly in the duodenum and proximal jejunum [13, 32]. In particular, GISTs in our series were more often located in the upper part of stomach (49, 49%), followed by the lower part of stomach (17, 17%), whereas most HPs were located in the lower part of stomach (25, 52.1%), followed by the duodenum (7, 14.6%). This distribution tendency of HP is consistent with many studies [2, 6, 20, 23, 31], and can be explained by “misplacement hypothesis”—HPs are fragments split from the main pancreas during embryonic rotation [30, 33].

Since HP often shows bright contrast enhancement similar to that of the main pancreas in arterial and/or venous phase [4, 20, 34], we calculated enhancement ratio of lesion to pancreas in both phases. Finally, some semi-quantitative and quantitative parameters showed significant differences in the degree of reinforcement between HP and GIST—HU lesion/pancreas A, HU arterial, DEAP, and enhancement grade, from which we inferred that HP might be more characteristically hyperenhanced in the arterial phase. Only HU lesion/pancreas A was included in both models as an independent predictor of HP, and when the ratio is greater than 0.805, the lesion is more likely to be diagnosed as HP. Meanwhile, we also deduce that the ratio of HPs to pancreas in portal venous phase was closer to 1 (Table 2, median values of HP and GIST were 1.03 and 0.90, respectively), which requires more studies to validate further.

Consisted of pancreatic acini, ductal components at different proportions, HP has three subtypes in histologic specimens—acini-dominant, duct-dominant and mixed type [9]. Based on above typing, we further classified the lesions into three types on imaging—solid-dominant, cystic-dominant and mixed type. Li et al. [20] removed the complete cystic HPs, in fact there were also 2 cases of cystic GIST in our study, and no statistical differences was found in image type between the two lesions finally. Other CT features, including ill-defined border, microlobulated appearance [30], presence of low intralesional attenuation and enhancement pattern [22, 30], were also associated with lobular architecture or dilated residual duct of HP [2, 4, 6], and were deduced by prior studies to be distinctive signs of HP. Yet they had not statistically significant difference or were just relevant predictors in our series. Due to HP likely extending to the muscularis propria or the entire wall of gastrointestinal tract [31, 35], we propose “peritumoral hypodensity line” to describe the relationship between the lesion and the peripheral gastrointestinal wall, which could reveal lesions as clear submucosal lesions or represented fat space between the extraluminal lesion and serosal layer (Fig. 2D). It was included in the RF model finally.

Duct-like structure was found to be a significant CT feature with secondary importance in the nomogram, which was referred to a central umbilication located at the mucosa of the lesion, corresponding to the rudimentary duct of the HP as seen in histologic specimens [30, 34]. T2-weighted MR images and magnetic resonance cholangiopancreatographic (MRCP) (Fig. 3B) images are best for confirming a dilated duct in HP, which is also referred to as the “ectopic duct” sign [4, 8, 11]. This sign was not included in the RF model. We infer that this sign, while unique to the ectopic pancreas, was relatively difficult to be observed. Only 7 of 48 HPs were detected this CT morphologic feature in this study, with 2 located in the lower part of stomach, 3 in the duodenum, and 2 in the jejunum or ileum, respectively. We inferred from this that the sign was easier to discern in the small intestine, similar to some previous reports [5, 30]. In addition, the trend in the age distribution of HP and GIST is consistent with most of the studies we’ve seen.

Our study has several limitations. First, the retrospective design may have introduced inherent selection bias, although our patients were enrolled from two institutions. Second, due to the long period of follow-up, different CT machines and protocols were used, which might influence the quantitative analysis. Third, due to the use of lesions LD less than 3 cm in the inclusion criterion, we excluded a great proportion of GISTs. Fourth, the size of our study population was small, especially for HP, and thus the stability of the model may be affected and the false-positive rates may increase.

## Conclusion

In brief, two convenient and efficient models based on CT signs was established using nomogram and machine learning method, and could be valuable for discriminating gastrointestinal HP from GIST in clinical practice. Both models have excellent diagnostic prediction performance, although prospective studies with larger sample sizes will be required to confirm these results.

## Data Availability

The datasets used or analyzed during the current study are available from the corresponding author on reasonable request.

## Abbreviations

HP: heterotopic pancreas
GIST: gastrointestinal stromal tumor
CT: computed tomography
HU: Hounsfield unit
LD: long diameter
SD: short diameter
RF: Random Forest
ROC: the receiver operating characteristic curve
AUC: the area under the curve
DCA: decision curve analysis
